# Intrinsic stability of magnetic anti-skyrmions in the tetragonal inverse Heusler compound Mn_1.4_Pt_0.9_Pd_0.1_Sn

**DOI:** 10.1038/s41467-019-13323-x

**Published:** 2019-11-22

**Authors:** Rana Saha, Abhay K. Srivastava, Tianping Ma, Jagannath Jena, Peter Werner, Vivek Kumar, Claudia Felser, Stuart S. P. Parkin

**Affiliations:** 1 Max Planck Institute of Microstructure Physics, Weinberg 2, 06120 Halle (Saale), Germany; 2 Institute of Physics, Martin Luther University, Halle-Wittenberg, 06120 Halle (Saale), Germany; 3 Max Planck Institute for Chemical Physics of Solids, Nöthnitzer Str. 40, 01187 Dresden, Germany

**Keywords:** Magnetic properties and materials, Electronic and spintronic devices

## Abstract

Magnetic anti-skyrmions are one of several chiral spin textures that are of great current interest both for their topological characteristics and potential spintronic applications. Anti-skyrmions were recently observed in the inverse tetragonal Heusler material Mn_1.4_Pt_0.9_Pd_0.1_Sn. Here we show, using Lorentz transmission electron microscopy, that anti-skyrmions are found over a wide range of temperature and magnetic fields in wedged lamellae formed from single crystals of Mn_1.4_Pt_0.9_Pd_0.1_Sn for thicknesses ranging up to ~250 nm. The temperature-field stability window of the anti-skyrmions varies little with thickness. Using micromagnetic simulations we show that this intrinsic stability of anti-skyrmions can be accounted for by the symmetry of the crystal lattice which is imposed on that of the Dzyaloshinskii-Moriya exchange interaction. These distinctive behaviors of anti-skyrmions makes them particularly attractive for spintronic applications.

## Abstract (web summary)

Magnetic anti-skyrmions—chiral spin textures that could find applications in spintronics—have been recently observed in inverse tetragonal Heusler Mn_1.4_Pt_0.9_Pd_0.1_Sn. Here, the authors observe anti-skyrmions in thin films of Mn_1.4_Pt_0.9_Pd_0.1_Sn over a wide range of temperature and magnetic fields.

## Introduction

Magnetic skyrmions (Sks) and anti-skyrmions (aSks) are topologically protected nanoscopic magnetic entities that can be stabilized in magnetic materials with broken inversion symmetry^1–6^. These innately chiral magnetic objects have been proposed as candidates for high-density racetrack memory, neuromorphic computing applications, and logic components^7–10^. Sks and aSks arise when there is an antisymmetric Dzyaloshinskii-Moriya exchange interaction (DMI)^11,12^ that competes with the more conventional Heisenberg exchange^9,13,14^. Anti-skyrmions have been reported to date only in the tetragonal inverse Heusler compound, Mn_1.4_Pt_0.9_Pd_0.1_Sn^14^, whereas distinct Sk textures have been realized in various crystal types, for example, Bloch Sks in several B20 compounds^15^, and Néel Sks in GaV_4_S_8_ and VOSe_2_O_5_^16,17^. Néel Sk bubbles have also been observed in magnetic multilayers via an interface DMI^18^. Although in the latter system it is clear that there will be a strong dependence of the Sk phase on the multilayer thickness, the situation for Sks and aSks stabilized by bulk DMI is less certain. The original discovery of Sks in the B20 compound MnSi by neutron diffraction on large crystals suggested extended Sk phases in three dimensions, in the form of arrays of extended skyrmion tubes along the applied magnetic field direction^3^, but more recent work suggests that these tubes are of finite extent^6,19,20^. Furthermore, the skyrmion phase in bulk B20 compounds is found only in a limited magnetic field (*B*)–temperature (*T*) region close to *T*_c_^3^. On the other hand, it has been found that in thin lamellae (~100 nm) made from single crystals of MnSi and FeGe the *B*–*T* stability region of the Sk phase is considerably enhanced^21,22^. The Sk phase diagram in the cubic B20 compounds has thus been found to be very sensitive to their thickness. Here we show, by contrast, that anti-skyrmions in Mn_1.4_Pt_0.9_Pd_0.1_Sn exhibit an extended *B*–*T* stability window even for lamella up to ~250-nm thick. We attribute this distinct property of anti-skyrmions to the anisotropic DMI vector whose symmetry is determined by the underlying *D*_2*d*_ crystal symmetry. Using micromagnetic simulations we compare the thickness-field dependent phase diagrams of cubic and *D*_2*d*_ systems that clearly shows the intrinsic stability of anti-skyrmions in the latter systems.

## Results

### Lorentz transmission electron microscopy measurements

**Fig. 1:**
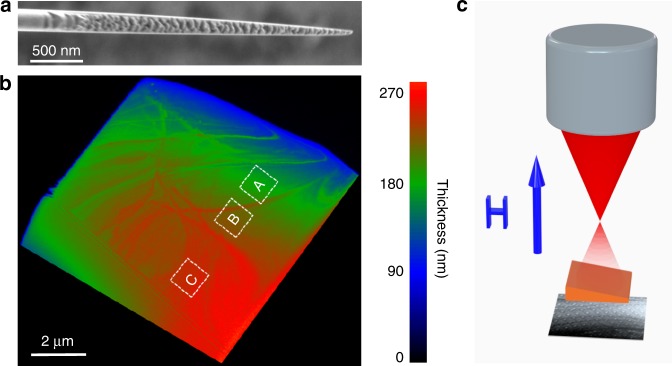
Wedge-shaped lamella of single crystalline Mn_1.4_Pt_0.9_Pd_0.1_Sn. **a** Cross-sectional view of a scanning electron microscopy (SEM) image of the wedge-shaped lamella. **b** 2D thickness map of the lamella obtained from electron energy loss spectroscopy (EELS) measurements. The three white dashed boxes indicate three regions of different thicknesses (A: 164 nm < *t* < 197 nm; B: 213 nm < *t* < 229 nm and C: 246 nm < *t* < 250 nm), that were used to construct thickness-dependent magnetic phase diagrams. Note that the length of the wedge in figure a can be estimated from the scale bar by taking into account a 36° tilt. **c** Schematic drawing of the LTEM experimental set up.

Specimens for Lorentz transmission electron microscopy (LTEM) studies were prepared from single crystal grains within bulk polycrystalline Mn_1.4_Pt_0.9_Pd_0.1_Sn using focused ion-beam milling (FIB). [001] oriented grains were identified by electron backscattering diffraction. Preparation of the bulk Mn_1.4_Pt_0.9_Pd_0.1_Sn samples is discussed in Methods. The LTEM specimens were fabricated in the form of wedge-shaped lamellae rather than a series of lamellae with distinct thicknesses, in order to avoid possible variations in sample properties such as stoichiometry and possible differences arising from the FIB preparation procedures. Figure 1a shows a scanning electron microscopy (SEM) image of the wedge-shaped lamella that was used (8 μm long and 7 μm wide) in these studies. Figure 1b shows its corresponding thickness profile obtained from electron energy loss spectroscopy (EELS)^23^. The [001] direction is perpendicular to the lamella. LTEM images were are shown for three representative regions A, B, and C, each 1 μm × 1 μm in area, with distinct thicknesses of ~164–197 nm, ~213–229 nm, and ~246–250 nm, as a function of temperature and field in Fig. 1b. These regions were mapped with respect to the edges of the specimen so that the same regions were imaged irrespective of thermal drifts and magnetic field induced rotations in the TEM (see Supplementary Fig. 1). These procedures are discussed in Supplementary Note 1. Figure 1c shows a schematic drawing of the LTEM experimental set up. We define the *z* direction to be along the column of the microscope. A double-tilt sample holder allowed for sample rotations about *x* and *y* and for the sample temperature to be varied from 100 to 365 K. The magnetic field was varied from −0.3 to + 2.3 T along the microscope column by adjusting the current in the objective lens.

The magnetic texture of the sample depends on the temperature and field. In addition to the aSk phase the sample can exhibit a helical magnetic texture^14^ where the helix direction is oriented along [100] or [010]. Both the aSk and helical phases depend on the temperature-field history so that careful procedures are needed to ensure coherent thickness-dependent magnetic phase diagrams. In these studies the LTEM measurements were carried out as follows. First, the sample is heated to the maximum possible temperature (365 K). Then the wedge is either zero-field cooled (ZFC) or field-cooled (FC) with 0.2 T along [001] to 100 K. Then, for the FC case, the field is set to zero. At this point (100 K) the ZFC state is completely in the helical phase and the FC state is in a mixed helical/aSk state. The sample is then tilted by ~10° about the [110] axis and a gradually increasing magnetic field is applied until the thinner regions A and B of the wedge are transformed into the ferromagnetic (FM) phase, whereas the thicker region C remains in a mixed FM/aSk state. Then the sample is tilted back to its original position whilst keeping the magnetic field constant. Then, LTEM images are acquired in successively decreasing magnetic fields in uniform steps of 0.016 T until the field is decreased to zero. The temperature of the sample is then increased and the same procedure is repeated. A lower field during the tilt process is required to get the aforementioned states at higher temperatures. These tilt field values are included in Supplementary Note 2. A sequence of measurements on wedge-lamella at 100, 150, 200, 250, and 300 K were thus made. Note that the application of a magnetic field on the tilted sample realizes an in-plane field component that facilitates the stabilization of the aSk lattice^14^.

**Fig. 2:**
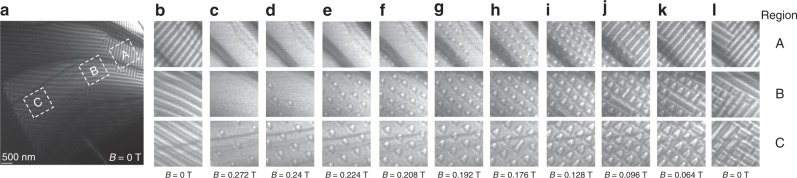
LTEM images of the wedge-shaped lamella at 300 K versus magnetic field. **a** LTEM image of the lamella at zero magnetic field immediately after warming from 250 K. **b**–**l** Sequence of LTEM images recorded after the field was first increased to 0.272 T from zero field and then decreased gradually to zero field for three regions (A, B, and C) of the lamella shown as the white dashed boxes in (**a**). Note that in region A bending contours are clearly visible (e.g., in **c**–**f**). The size of each image in (**b**–**l**) is 1 × 1 μm^2^.

**Fig. 3:**
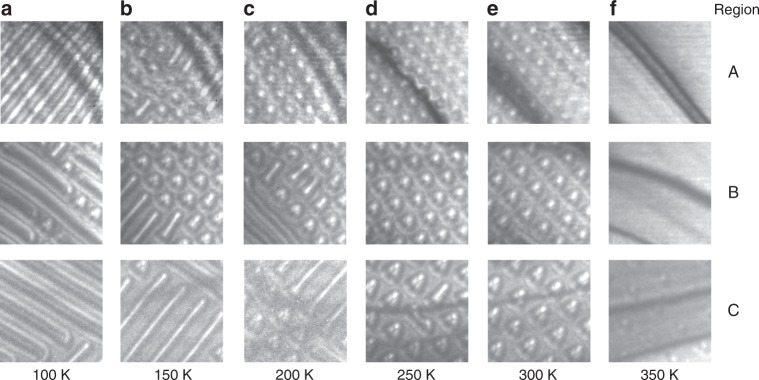
LTEM images of the wedge-shaped lamella at different temperatures. The measurments are performed in the presence of a perpendicular magnetic field of 0.16 T. **a**–**f** LTEM images recorded for three regions (A, B, and C) of the lamella shown as the white dashed boxes in Fig. 2a. The size of each image is 1 × 1 μm^2^.

A typical set of LTEM images taken at 300 K are shown in Fig. 2. Figure 2a shows the LTEM image of the wedge in zero magnetic field after warming from 250 K. A helical phase is observed throughout the wedge whose direction of propagation is along either [010] or [100]. Figure 2b shows the magnified regions, A, B, and C cropped from the image in Fig. 2a. Figure 2c–l shows these same regions as the magnetic field is reduced according to the above protocol. For example, Fig. 2c is obtained after the sample was tilted by ~10° about [110], a field of 0.272 T was applied, and then the sample was tilted back to a zero-tilt angle. In this field a FM phase is found in regions A and B, while region C is partly FM and partly aSk. An aSk phase gradually appears throughout the wedge as the field is reduced (Fig. 2c–i): at 0.24 T in region C, 0.208 T in region B, and 0.176 T in region A. Upon further reduction of the field (0.096–0.064 T, Fig. 3j, k), the helical magnetic phase appears first in region A, a mixed phase of helical and aSks appears in region B, and the aSk phase remains intact in region C. Finally, when the field is reduced to zero (Fig. 2), the helical magnetic states appears throughout the sample. Clearly, as the thickness of the lamella is increased, the field at which the field-polarized state transforms to the aSk phase increases, while that of the aSk to helix phase decreases. Thus, we find that the aSk region is somewhat enlarged as the lamella thickness is increased.

In Fig. 3 we show the temperature-dependent evolution of the LTEM images for the three thickness regions for a magnetic field of 0.16 T for data taken using the same protocols discussed above. At 100 K the helix state is predominant for all thicknesses, except for a few isolated aSks in region B (Fig. 3a). At 150 K the aSk lattice coexists with the helical phase in regions A and B, while in region C the helical phase coexists with a few isolated aSks (Fig. 3b). At 200 K an aSk lattice is found in A, while in B and C an aSk lattice coexists with the helical phase (Fig. 3c). At 250 and 300 K the aSk lattice appears in all three thickness regions (Fig. 3d, e). At 350 K the aSk lattice is found only in C, while A and B are fully field-polarized (Fig. 3f).

**Fig. 4:**
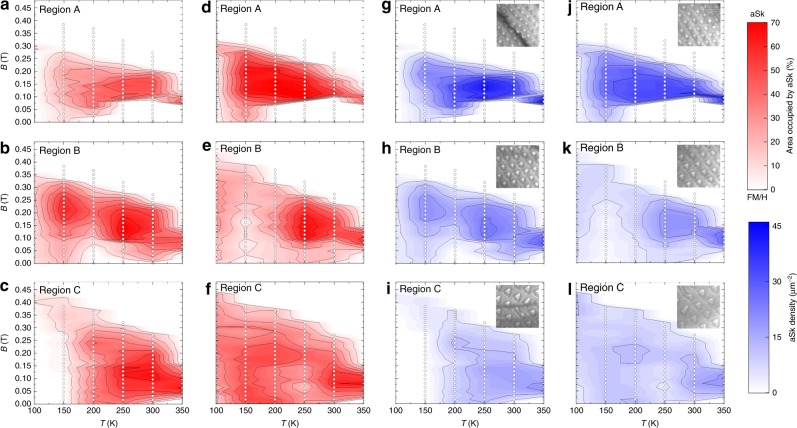
Magnetic phase diagram of the wedge-shaped lamella. The experiments are performed after zero-field-cooling (ZFC) and field-cooling (FC), as a function of temperature (*T*) and perpendicular magnetic field (*B*), for three distinct regions A, B, and C. The area occupied by the aSk phase is shown in (**a**–**c**) for ZFC and in (**d**–**f**) for FC. The aSk density is shown in (**g**–**i**) for ZFC and in (**j–l**) for FC. The insets (1 × 1 μm^2^) in (**g**–**l**) show LTEM images of the aSk lattice recorded in 0.176 T at 250 K. Note that 70% corresponds to a pure aSk lattice (for details see SI). The white dots in each phase diagram indicate the magnetic field and temperature where LTEM images were collected.

The temperature and magnetic field dependence of the area of the sample occupied by aSks and the aSk density in regions A, B, and C are shown in Fig. 4 (see Supplementary Fig. 2 and Supplementary Note 3 for details of the analysis procedures used). The aSk phase is stable over a wide temperature range to above room temperature and magnetic field for all thicknesses studied. Qualitatively, the aSk stability window is similar at all thicknesses, with a tendency toward a larger window in thicker lamellae. There is a more pronounced difference between the ZFC and FC processes at lower temperatures. For ZFC there are only a few isolated aSks in all thickness regions and magnetic fields at 100 K (Fig. 4a–c) whereas for the FC process (Fig. 4d–f) many more aSks are observed at the same temperature.

The inset of Fig. 4g–i (ZFC) and 4j–l (FC) shows typical LTEM images of the aSk phase in regions A, B, and C at 250 K in a field of 0.176 T. From these images, we find that the size of the aSks increase with lamella thickness, resulting in a decrease in the aSk density. These observations are quite distinct from those reported for the Sk phase in B20-type cubic chiral magnets^21,22^, where the Sk size does not vary with lamella thickness and the *B*–*T* Sk phase stability window shrinks as the thickness of the sample is increased.

## Discussion

The most important conclusion that we can draw from our extensive LTEM measurements is that the aSk phase in Mn_1.4_Pt_0.9_Pd_0.1_Sn can be stabilized by appropriate field-temperature protocols over a wide range of temperature and field and which has a weak dependence on thickness of the lamella. This is in sharp contrast to the B20 compounds. To understand the origin of the distinctive thickness-dependent aSk and Sk phase diagrams, we consider a microscopic model using the following Hamiltonians for the *D*_2*d*_ and B20 systems, respectively,

1 $$H_{B20} =	 \, - J\mathop {\sum}\limits_{\mathbf{r}} {\vec {\mathbf{S}} _{\mathbf{r}}} \cdot \left( {\vec {\mathbf{S}} _{{\mathbf{r}} + a\hat {\mathbf{x}}} + \vec {\mathbf{S}} _{{\mathbf{r}} + a\hat {\mathbf{y}}} + \vec {\mathbf{S}} _{{\mathbf{r}} + a\hat {\mathbf{z}}}} \right) - {\vec{\mathbf{B}}}\mathop {\sum}\limits_{\mathbf{r}} {\vec {\mathbf{S}} _{\mathbf{r}}}\\ 	 - D_{B20}\mathop {\sum }\limits_{\mathbf{r}} \left( {\vec {\mathbf{S}} _{\mathbf{r}} \times \vec {\mathbf{S}} _{{\mathbf{r}} + a\hat {\mathbf{x}}} \cdot {\hat{\mathbf{x}}} + \vec {\mathbf{S}} _{\mathbf{r}} \times \vec {\mathbf{S}} _{{\mathbf{r}} + a\hat {\mathbf{y}}} \cdot \hat {\mathbf{y}} + \vec {\mathbf{S}} _{\mathbf{r}} \times \vec {\mathbf{S}} _{{\mathbf{r}} + a\hat {\mathbf{z}}} \cdot \hat {\mathbf{z}}} \right)$$

2 $$H_{D_{2d}} =	 - J\mathop {\sum}\limits_{\mathbf{r}} {\vec {\mathbf{S}} _{\mathbf{r}}} \cdot \left( {\vec {\mathbf{S}} _{{\mathbf{r}} + a\hat {\mathbf{x}}} + \vec {\mathbf{S}} _{{\mathbf{r}} + a\hat {\mathbf{y}}} + \vec {\mathbf{S}} _{{\mathbf{r}} + a\hat {\mathbf{z}}}} \right) - {\vec{\mathbf{B}}} \cdot \mathop {\sum}\limits_{\mathbf{r}} {\vec {\mathbf{S}} _{\mathbf{r}}}\\ 	 - D_{D_{2d}}\mathop {\sum }\limits_{\mathbf{r}} \left( { - \vec {\mathbf{S}} _{\mathbf{r}} \times \vec {\mathbf{S}} _{{\mathbf{r}} + a\hat {\mathbf{x}}} \cdot \hat {\mathbf{x}} + \vec {\mathbf{S}} _{\mathbf{r}} \times \vec {\mathbf{S}} _{{\mathbf{r}} + a\hat {\mathbf{y}}} \cdot \hat {\mathbf{y}}} \right) \qquad \qquad$$

where *J* is the exchange stiffness, *B* is the external field, *D*_*B*20_ and $D_{D_{2d}}$ are the respective DMI coefficients according to the B20 and *D*_2*d*_ systems, $\vec {\mathbf{S}} _{\mathbf{r}}$ is the magnetization at position $\vec {\mathbf{r}}$, whereas $\vec {\mathbf{S}} _{{\mathbf{r}} + a\hat {\mathbf{x}}},\vec {\mathbf{S}} _{{\mathbf{r}} + a\hat {\mathbf{y}}},\vec {\mathbf{S}} _{{\mathbf{r}} + a\hat {\mathbf{z}}}$ are the magnetization values of the adjacent cells in the *x*, *y*, and *z* directions. In each case we include the same Heisenberg exchange and Zeeman energy terms, but the DMI terms have distinct symmetries according to the respective B20 and *D*_2*d*_ crystal structures^1^. For the B20 system the DMI vector is identical along *x*, *y*, and *z*, whereas in a *D*_2*d*_ system, the DMI vectors along *x* and *y* have the same magnitude but opposite sign and the DMI vector along *z* is zero. Note that the simulations are innately 3-dimensional with both Heisenberg and DMI exchange interactions included along *x*, *y*, and *z*. Note that we neglect the influence of dipolar interactions that have usually not been considered in exploring the phase stability of skyrmions^24,25^. We also do not include magnetic anisotropy since this will not change our major conclusions.

In B20-type materials which are cubic, the component of the DMI vector along the thickness direction should give rise to a chiral modulation of the magnetization through the thickness of a layer that leads to strong dependence of the phase diagram on thickness as theoretically discussed^19,24,26,27^. Indeed, in both MnSi and FeGe it has been found experimentally that the Sk phase *B*–*T* stability region shrinks with increasing thickness to a small region by ~100 nm^21,22^. Recently, it has been observed in both FeGe single crystals and thin films that, as the thickness becomes larger, the modulation increases until a critical thickness at which the Sk-tube structure breaks up into chiral bobbers^19,28^. By contrast, in the *D*_2*d*_ system, the component of the DMI vector along the [001] is zero so that no modification of an  aSk is allowed along the thickness of [001] oriented lamellae. Thus, there should be a much weaker dependence of the *B*–*T* phase stability of aSk on thickness.

We have performed micromagnetic simulations based on the Hamiltonian introduced above to explore and compare the thickness-dependent behavior for aSks and Sks (see Methods for details). Typical dependences of the B20 Sk and aSk structures are shown in Supplementary Fig. 3 for two distinct layer thicknesses. In each case the detailed magnetic structure is shown at several representative *z* values (see Supplementary Note 4). In the B20 material, there is a clear chiral modification of the Sk-tube along the *z* direction (Supplementary Fig. 3a–c). As the thickness goes beyond a critical value, the Sk-tube breaks down into chiral bobbers (Supplementary Fig. 4a–e). This results in a breakdown of the Sk phase that can most clearly be seen in the thickness averaged magnetization, as shown in Supplementary Figs. 3–6. However, in the *D*_2*d*_ system, there is no change in the aSk structure along *z*, so that a clear aSk phase can be seen in the thickness averaged magnetization (Supplementary Figs. 3h and 4l). A similar behavior also applies to the helical phase (Supplementary Fig. 5).

We note that in these simulations neither a significant change in the aSk size nor in the helix period is found. We attribute the changes in these quantities that we have observed experimentally, as mentioned above, to magnetic dipole interactions.

**Fig. 5:**
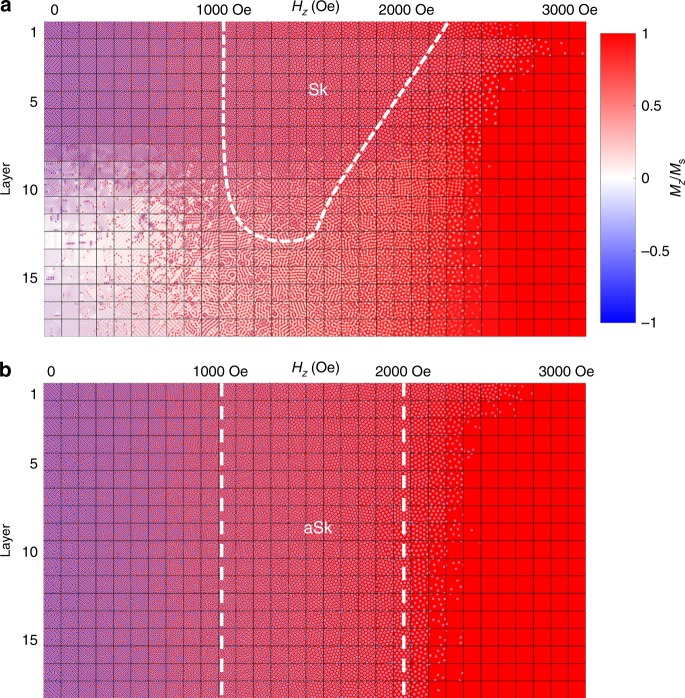
Micromagnetic simulations of the phase diagrams of B20 and *D*_2*d*_ systems. A table of micromagnetic simulations of the magnetic texture for various magnetic fields (*B*) and thicknesses with increasing *B* along the abscissa and increasing thickness along the ordinate. For each calculated image in the table, the averaged *M*_z_ along the thickness direction is plotted, where the color indicates the amplitude of *M*_z_ normalized by the saturation magnetization *M*_s_. Red/blue corresponds to *M*_z_/*M*_s_ = +1/−1. **a** In a B20-type material, the field region of the skyrmion phase decreases as the thickness increases and finally disappears. **b** In a *D*_2*d*_-type material, the field region of the anti-skyrmion phase is unchanged as the thickness is increased. The simulations included in the Table are shown in higher magnification in the Supplementary Information.

Typical micromagnetic simulations of the field dependent behavior of the *z* component of the magnetization averaged along the thickness direction are summarized in Fig. 5 as the thickness of the lamella is varied. Clearly, the Sk region in the B20 material decreases with increasing thickness until it vanishes, as found previously^19,24,26,27^. On the other hand, the aSk region is preserved to large thicknesses, consistent with our experimental results.

In conclusion, we have experimentally determined the thickness-dependent magnetic phase diagram of the aSk phase in an inverse tetragonal Heusler material with *D*_2*d*_ symmetry. We observe that the field-temperature stability window of the aSk phase is largely insensitive to the sample thickness in contrast to earlier studies of B20 materials. Using micromagnetic simulations we show that this important difference can be attributed to the underlying symmetry of the corresponding Dzyaloshinskii-Moriya exchange interaction.

## Methods

### Experiment

The polycrystalline sample of Mn_1.4_Pt_0.9_Pd_0.1_Sn was prepared by first arc-melting in a high-purity argon atmosphere stoichiometric amounts of the constituent elements to form an ingot. The as-prepared ingots were annealed for 1 week at 1073 K and subsequently quenched in an ice-water mixture to obtain the final material. For the transmission electron microscopy (TEM) investigation, we have prepared a wedge-shaped lamella by means of a Ga focused ion-beam (FIB) milling apparatus [FEI Nova Nanolab 600 SEM/FIB operating at 30 keV ion-beam energy]. The thickness of the lamella was determined by electron energy loss spectroscopy (EELS)^23^. For this purpose we have determined the local thickness, *t*, from the relationship, *t* = *λ* ln (*I*_t_/*I*_0_), where *λ* is the total inelastic mean free path of electrons, *I*_t_ and *I*_0_ are the total and zero-loss intensities in the EELS spectrum, respectively. The sample has a thickness that varies from ~90 to ~250 nm. TEM investigations were carried out with an aberration-corrected, high-resolution transmission electron microscope [FEI TITAN 80-300] operated at an acceleration voltage of 300 keV using a GATAN double-tilt sample holder allowing for variable temperatures between 100 and 365 K. For the investigation of anti-skyrmions, TEM experiments were performed in the conventional Lorentz TEM (LTEM) mode. An in situ vertical magnetic field was applied to the wedge-shaped lamella by applying currents in the objective lens. Thereby, in-plane magnetic fields were realized by tilting the sample using a double-tilt sample holder (maximum tilting up to ~± 30°).

### Micromagnetic simulations

Micromagnetic simulations were performed by OOMMF^29,30^. In order to have a controlled comparison, the same parameters are used for both the B20 and *D*_2*d*_ systems. We use an exchange stiffness constant *J* = 1.2 × 10^−10^ Jm^−1^, a saturation magnetization *M*_s_ = 445 kAm^−1^, and the DMI, $\it D_{B20} = D_{D_{2{\mathrm{d}}}} = 6 \, \times 10^{ - 3}\,{\mathrm{Jm}}^{ - 2}$, where the OOMMF code^29,30^ was modified to allow for distinct DMI vector components in three dimensions. These values are taken from our previous work^14^. Due to the large calculation load, we chose a cell size of 40 nm × 40 nm × 40 nm. The total simulated volume is 2000 nm × 2000 nm × 40 nm × *N*_Layer_, where *N*_Layer_ is the number of layers along the thickness direction. We use in-plane periodic boundary conditions. In order to obtain the global ground state, we start the calculation at an extremely high temperature to ensure an absence of any stable magnetic structure. Then the temperature is gradually decreased to zero, where the global ground state is stabilized. We use OOMMF’s extend class of UHH_ThetaEvolve, in which the calculation temperature starts at 5,000,000 and decreases in steps of 1,000,000. For each temperature step 50,000 calculation steps are performed at a fixed time step of 1 ps. Finally, at zero temperature, the calculation stops when the max d*m*/d*t* reaches 0.0001 deg/ns. It should be noted that the temperature steps are different from our previous work^14^. Previously, we used a starting temperature of 200,000 and a temperature step of 50,000. The goal is to overcome the local energy barriers that arise from random starting topology which cannot be overcome at low temperatures. The use of extremely high temperatures in this work ensures a global ground state which more accurately gives the thickness-dependent phase diagram. In order to perform thickness-dependent simulations of arrays of Sks and aSks in the B20 and *D*_2*d*_ systems, the simulations must be carried out over large length scales in both the in-plane and thickness directions.

The size of the minimum cell size (over which the magnetization is fixed) was increased to make the simulations computationally tractable. Exemplary simulations for cell sizes of 40, 20, and 10 nm are shown in Supplementary Fig. 7 and discussed in Supplementary Note 5. These show the robustness of the phase diagram, the main goal of the simulations, to the cell size, but exact details of the magnetic order are affected.

## Supplementary information

Supplementary Information

## Data Availability

The data that support the findings of this study are available from the corresponding author upon request.
