# Supplementary material for: Intrinsic stability of magnetic anti-skyrmions in the tetragonal inverse Heusler compound Mn1.4Pt0.9Pd0.1Sn
Source: Nat Commun. 2019 Nov 22;10:5305. doi: 10.1038/s41467-019-13323-x (PMC6874587; doi:10.1038/s41467-019-13323-x)
Supplement: Supplementary file 1 — Supplementary Information [file 41467_2019_13323_MOESM1_ESM.pdf]

Supplementary Information

Intrinsic stability of magnetic anti-skyrmions in the tetragonal inverse Heusler compound $Mn_{1.4}Pt_{0.9}Pd_{0.1}Sn$

Rana Saha[1], Abhay K. Srivastava[1,2], Tianping Ma[1,2], Jagannath Jena[1,2], Peter Werner[1], Vivek Kumar[3], Claudia Felser[3], and Stuart S. P. Parkin[1,2*]

[1]Max Planck Institute of Microstructure Physics, Weinberg 2, 06120 Halle (Saale), Germany

[2]Institute of Physics, Martin Luther University, Halle-Wittenberg, 06120 Halle (Saale), Germany

[3]Max Planck Institute for Chemical Physics of Solids, Nöthnitzer Str. 40, 01187 Dresden, Germany

*email: stuart.parkin@mpi-halle.mpg.de

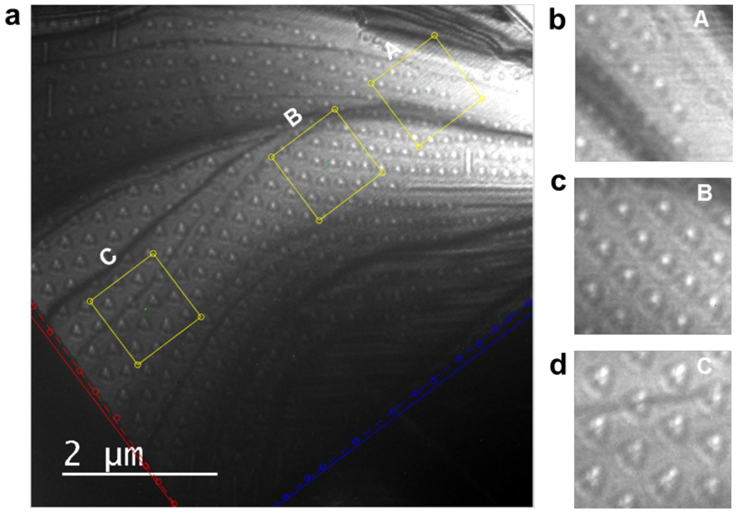

Supplementary Figure 1 Selection of three different thickness regions. **a** LTEM image at 300 K in the presence of a magnetic field of 0.192 T. The outlined boxes, A,B and C, correspond to regions with three different thicknesses (A: 164 nm < t < 197 nm; B: 213 nm < t < 229 nm and C: 246 nm < t < 250 nm). These regions were used to construct thickness-dependent magnetic phase diagrams. **b-d** magnified LTEM images of the three regions shown in a. Each image from b-d corresponds to an area of 1x1 μm^2.

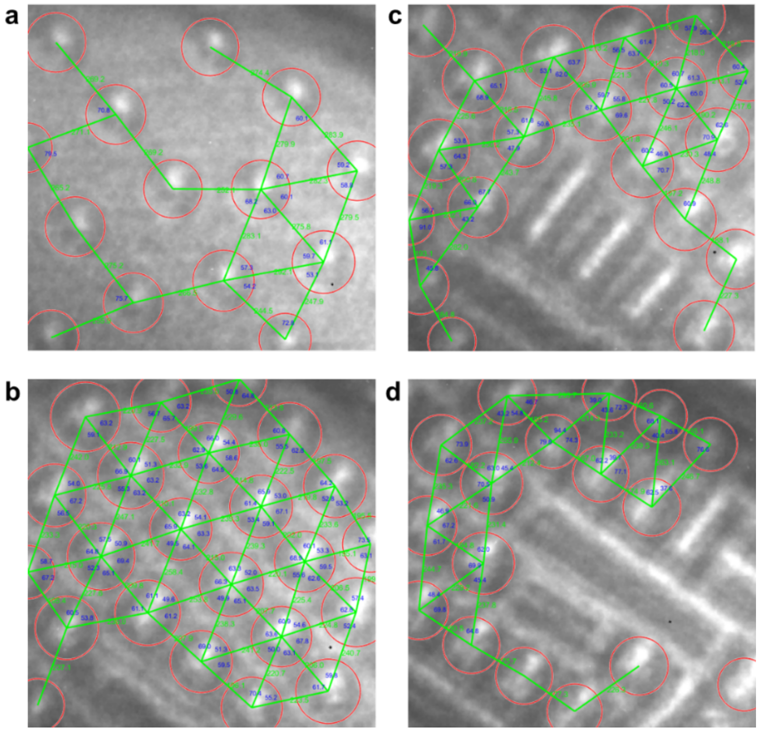

Supplementary Figure 2 Analysis of the area occupied by the aSk lattice. Analysis of typical LTEM images of the aSk lattice at 300 K at 4 different magnetic fields shows the area occupied by the aSk phase in these images to be: **a** 28 % in the FM + aSk phase at B = 0.224 T; **b** 53 % in the aSk phase at B = 0.128 T; **c** 40 % in the H + aSk phase at B = 0.112 T, and **d** 33% in the H + aSk phase at B = 0.048 T. These images correspond to region B (213 nm < t < 229 nm) in Fig. 2a. (H = helical; aSk = anti-skyrmion; FM = ferromagnetic). Each image from a-d corresponds to an area of 1x1 μm^2.

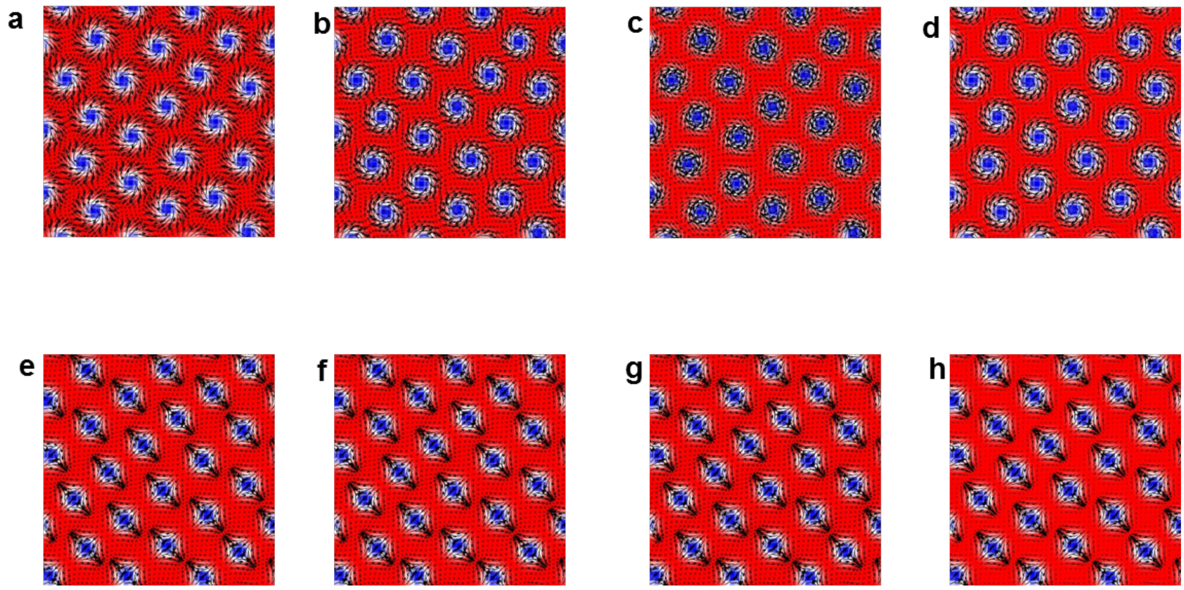

Supplementary Figure 3 Micro-magnetic simulations of 7 layers with B = 2000 Oe. **a-d** Magnetization profile of layer 1, 4, and 7 and average over all 7 layers in a B20-type system, where a clear variation from layer to layer is seen. **e-h** Magnetization profile of layer 1, 4, 7 and average over all 7 layers for a D_{2d} system, where every layer is found to have the same magnetization profile. A clear Sk/aSk lattice is shown in both systems. Color indicates the out-of-plane component of the magnetization and black arrows indicate the in-plane magnetization direction.

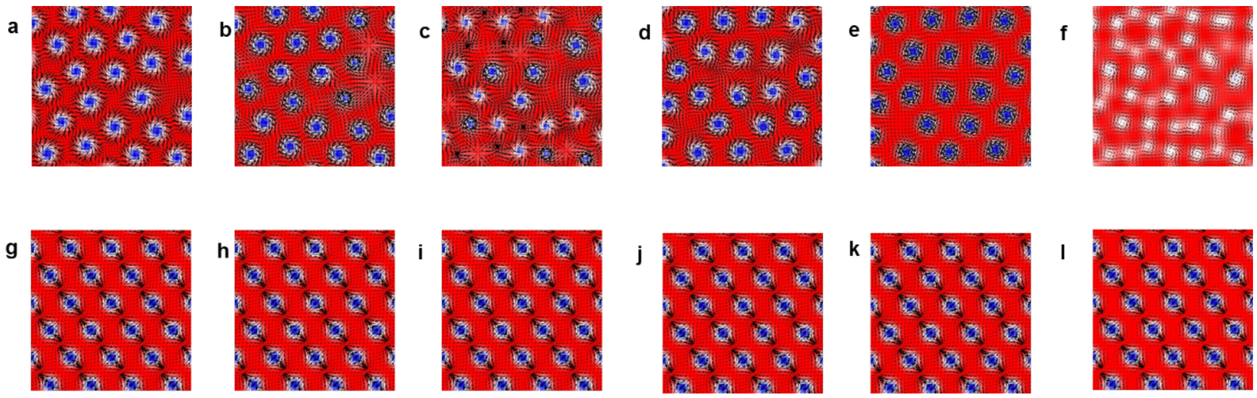

Supplementary Figure 4 Micro-magnetic simulations of 13 layers with B = 2000 Oe. **a-f** Magnetization profile of layers 1, 4, 7, 10, and 13 and average over all 13 layers for a B20 type system, where a clear variation from layer to layer is found. **g-l** Magnetization profile of layers 1, 4, 7, 10, and 13 and average over all 13 layers in a D_{2d} type system, where each layer has the same magnetization profile. The Sk lattice is broken up in the B20 structure but a clear aSk lattice is seen in the D_{2d} system. Color indicates the out-of-plane component of the magnetization and black arrows indicate the in-plane magnetization direction.

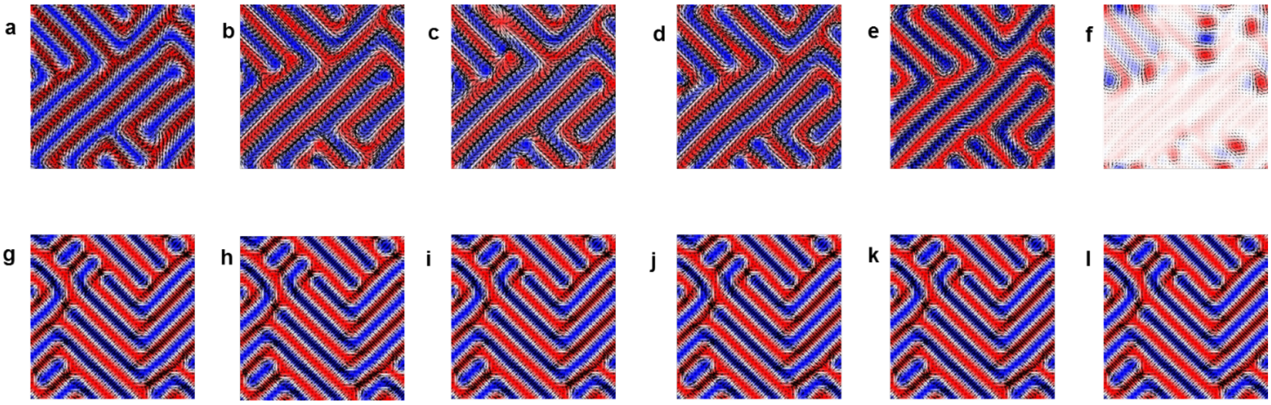

Supplementary Figure 5 Micro-magnetic simulations of 13 layers with B = 300 Oe. **a-f** Magnetization profile of layers 1, 4, 7, 10, and 13 and average over all 13 layers for a B20-type system. **g-l** Magnetization profile of layers 1, 4, 7, 10, and 13 and average over all 13 layers for a D_{2d} system. Color indicates the out-of-plane component of the magnetization and black arrows indicate the in-plane magnetization direction.

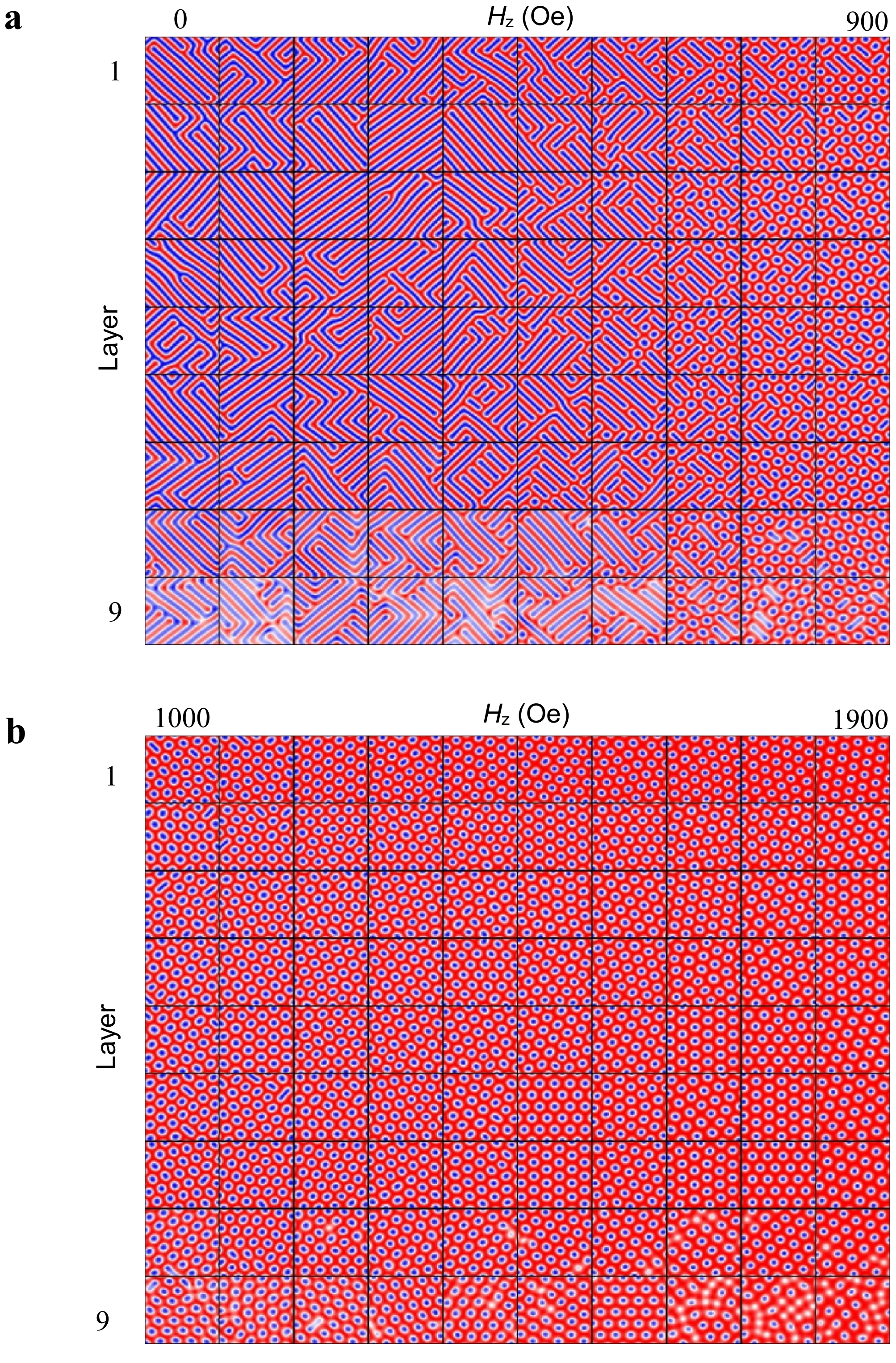
a
0
H_z (Oe)
900
1
Layer
9
b
1000
H_z (Oe)
1900
1
Layer
9

c

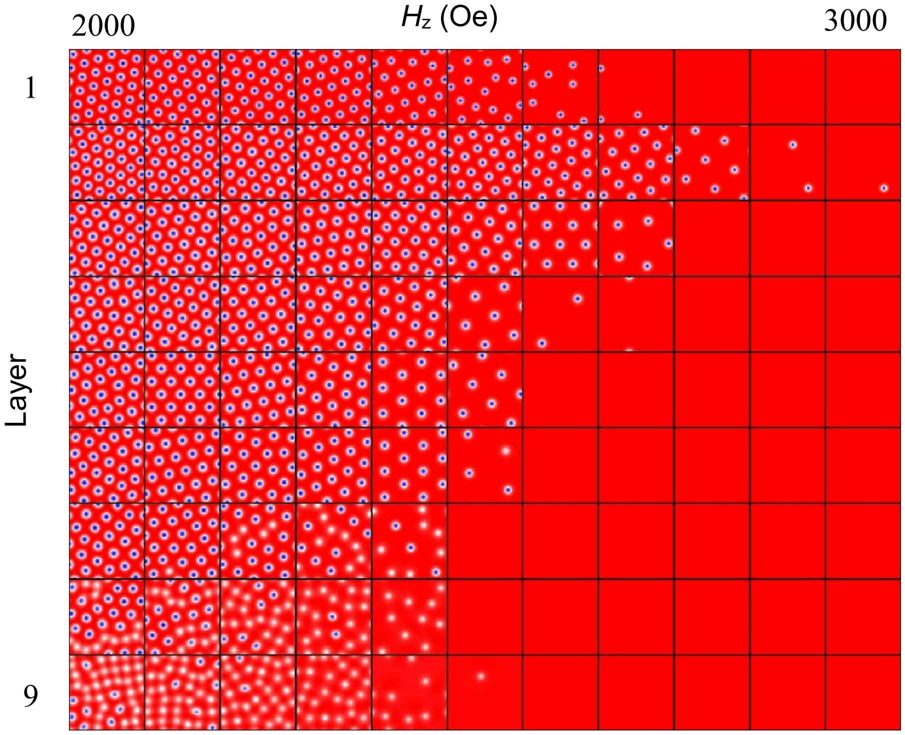

d

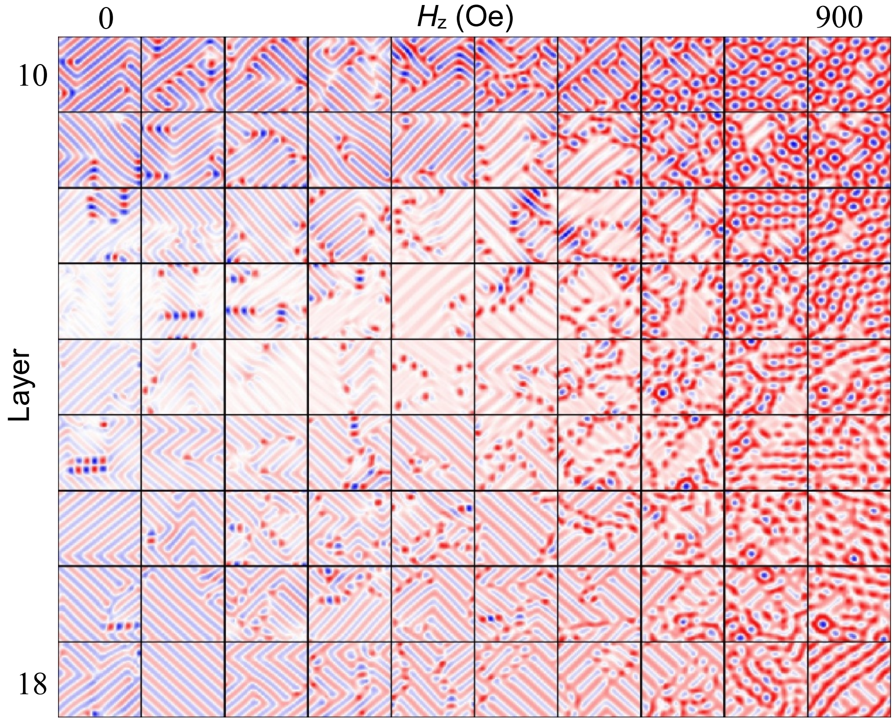

e

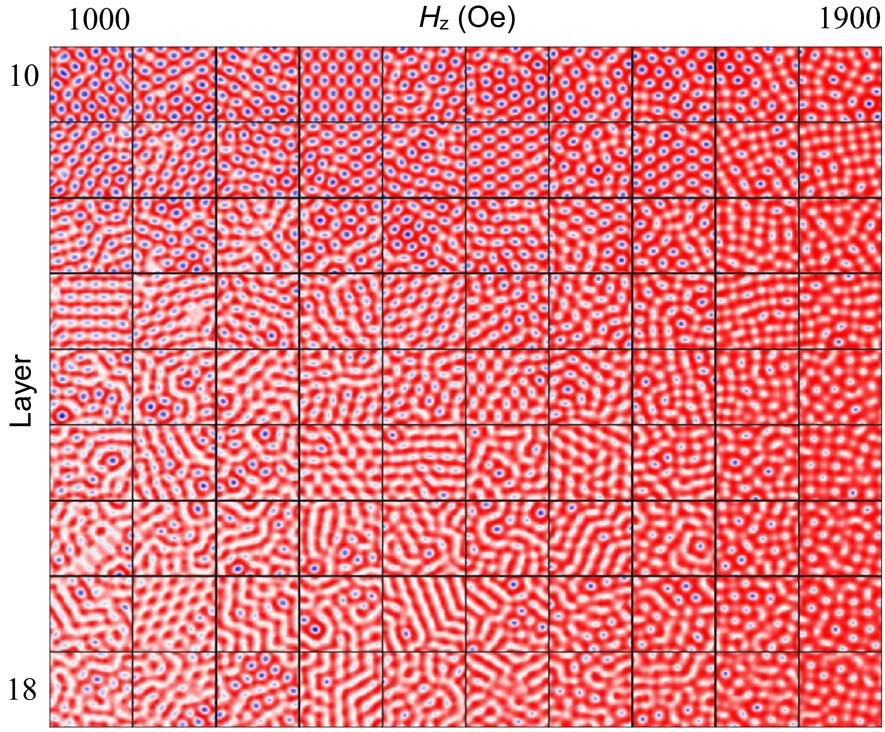

f

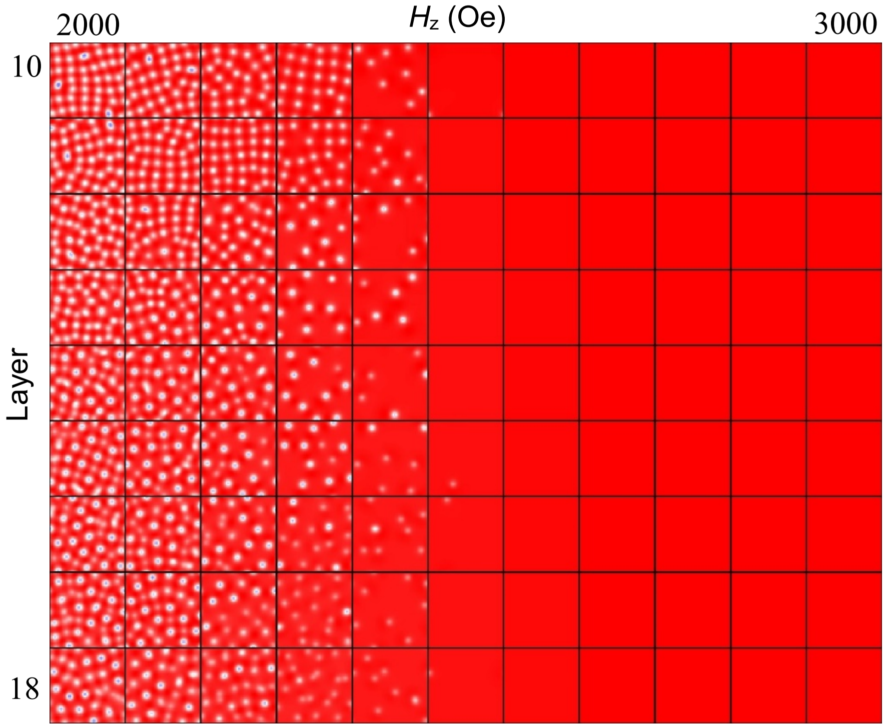

g

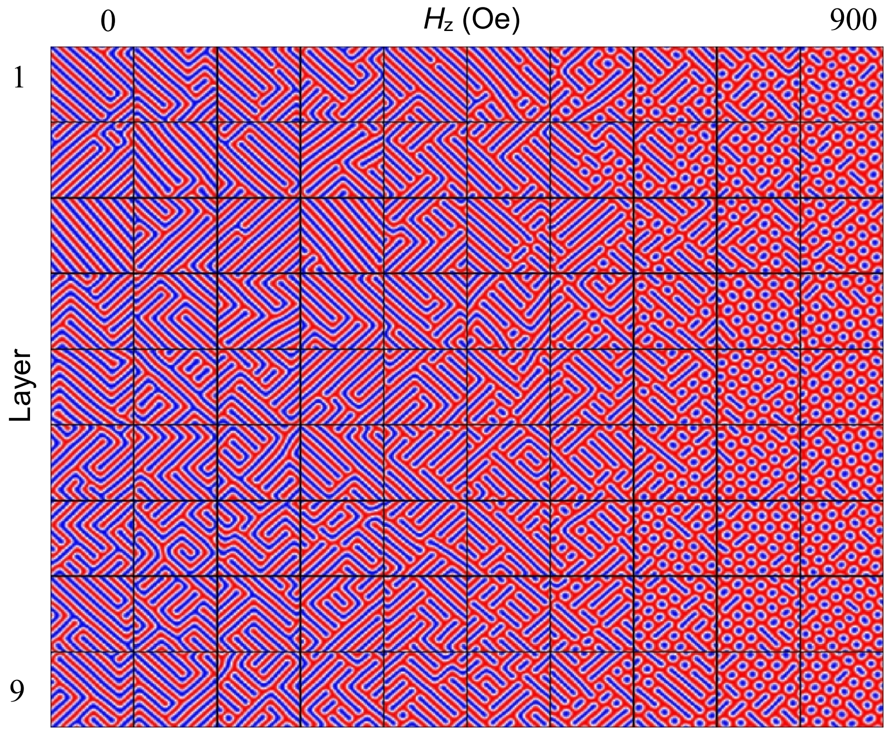

h

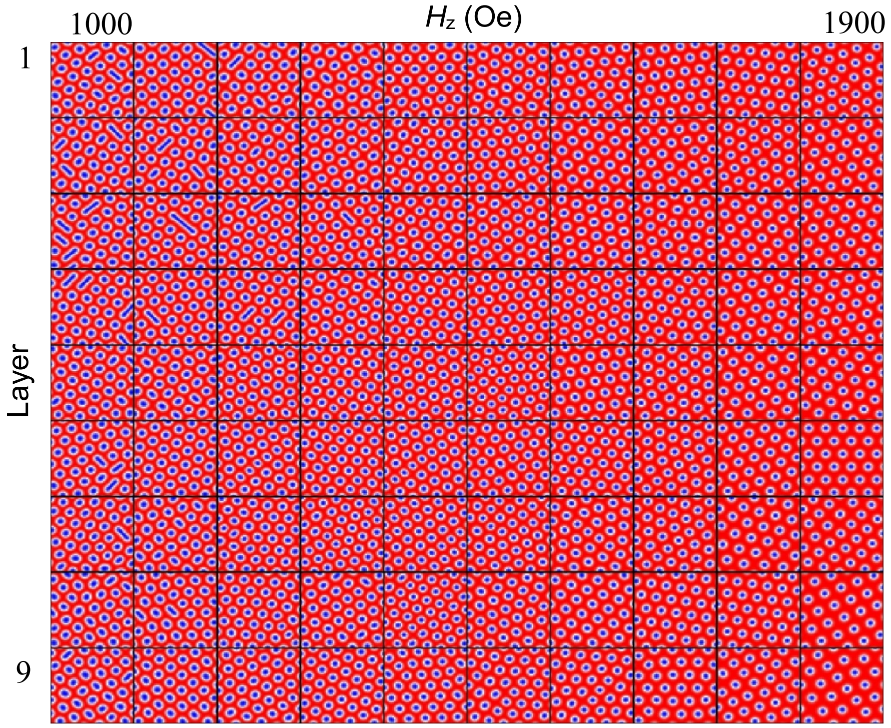

i

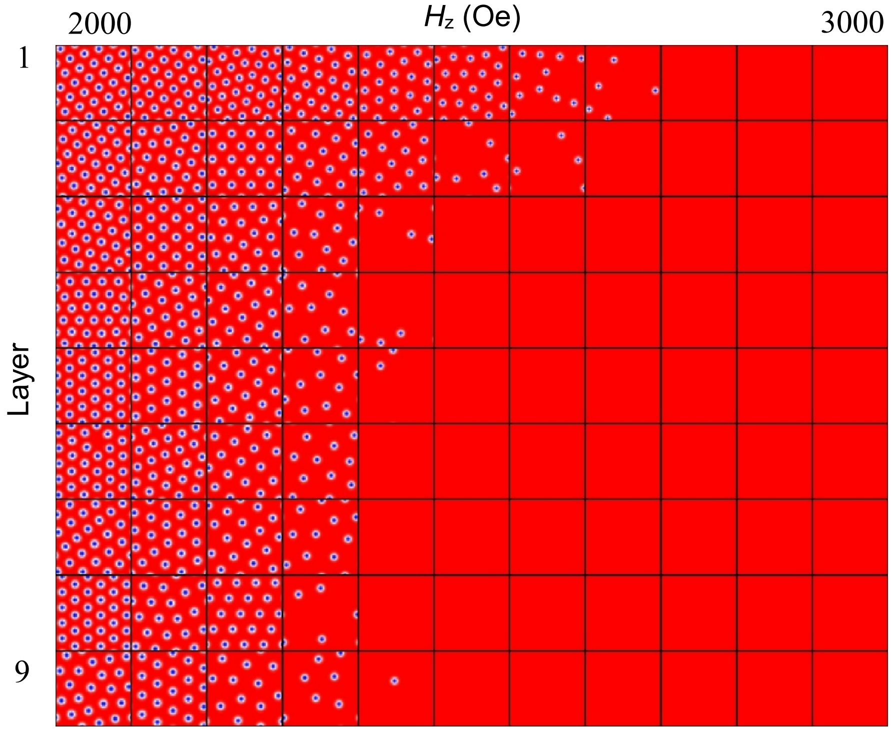

j

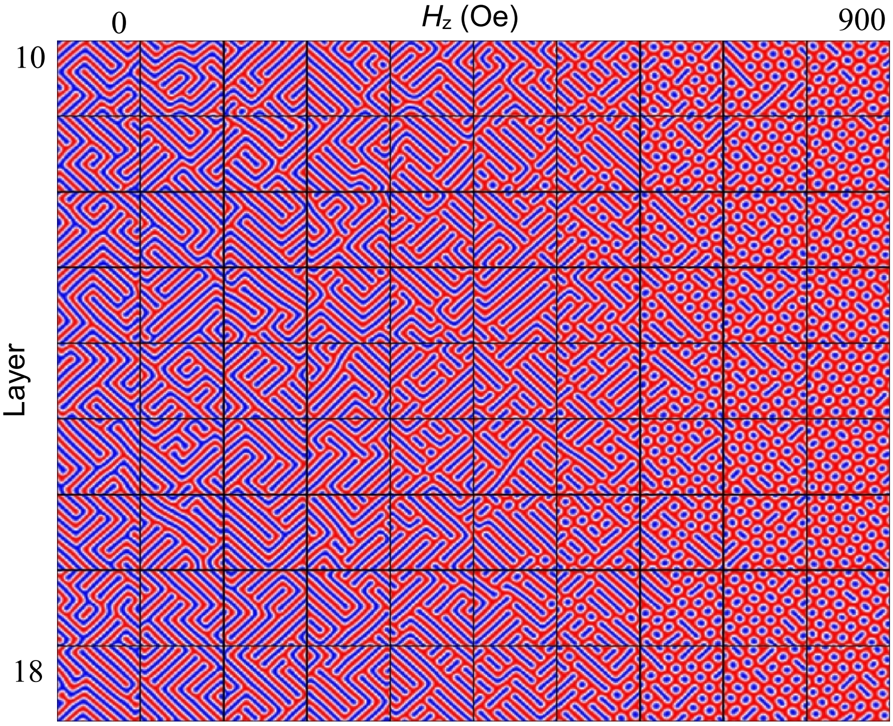

k

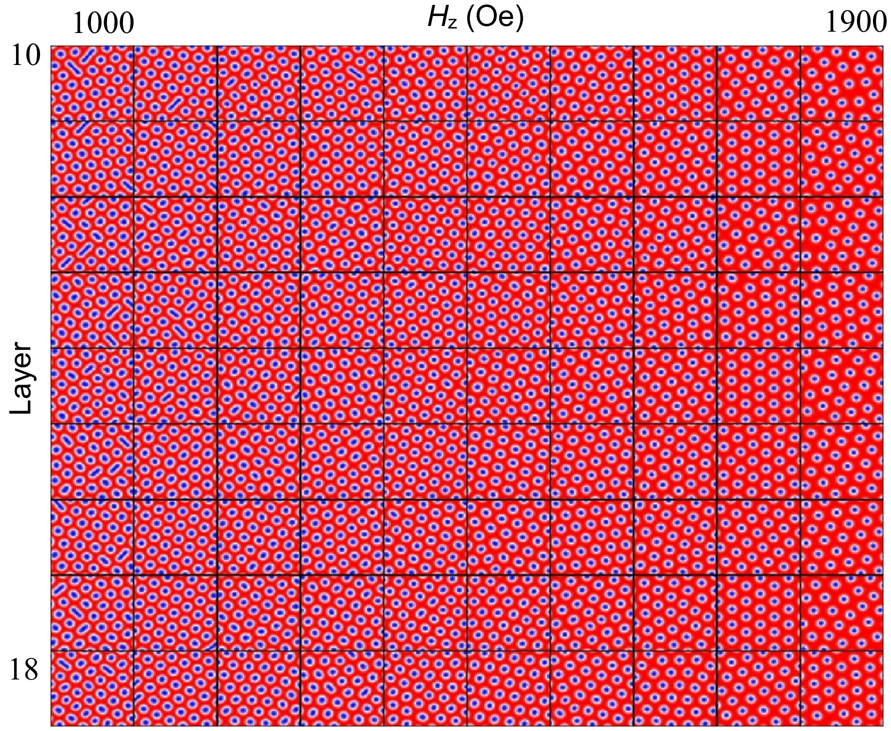

l

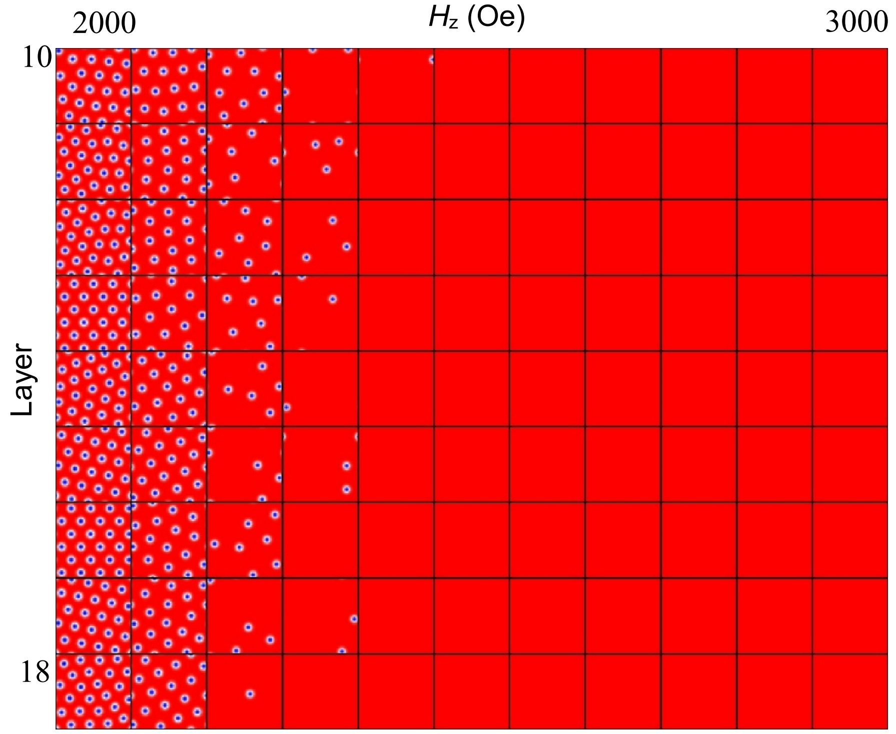

Supplementary Figure 6 Micromagnetic simulations of the phase diagrams of **a-f** B20 and **g-l** D_{2d} systems. These figures are an enlarged version of Fig. 5 of the main text. Here, as an aid to the reader, we show enlarged versions of Fig. 5a and 5b of the main text.

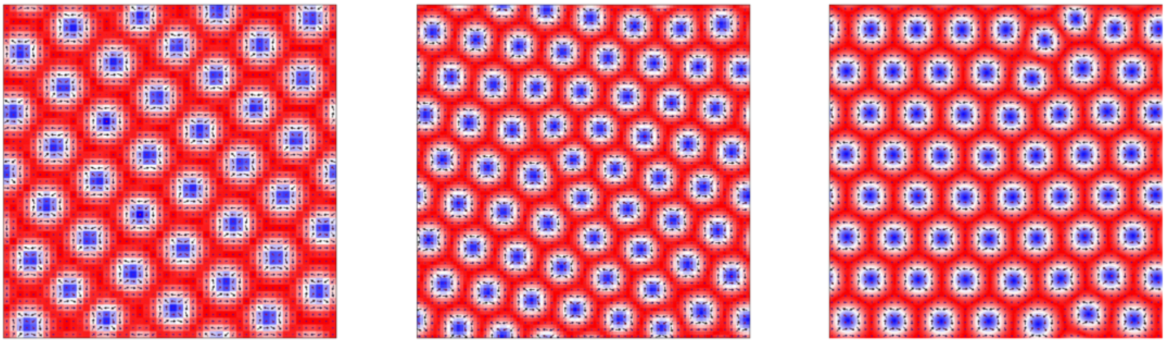

$\Delta = 40\ nm$ $\Delta = 20\ nm$ $\Delta = 10\ nm$

Supplementary Figure 7 Simulation results for different cell sizes. Parameters used: 2000 nm×2000 nm×1 nm size with $\Delta\times\Delta\times1$ nm cell, A = 120 pJ/m, K_{eff} = 0 MJ/m^3, M_s = 445 kA/m, H_z = 1800 Oe.

Supplementary Note 1: Selection of LTEM imaged regions of different thicknesses

As the temperature and field are varied the lamella may drift or rotate slightly. To ensure that the same regions are analyzed, image processing software is used, as illustrated in Supplementary Figure 1, to locate the same region in each LTEM image. The detailed steps are as follows:

a. Load the LTEM image, as well as the necessary parameters (e.g. pixel to distance ratio)
b. Locate the boundaries of the nearly rectangular lamella (shown as red/blue circles). Linear fitting of the boundaries to give two boundary lines (shown as red/blue dashed lines).
c. The intersection of the two boundary lines is set as the origin. Two perpendicular lines (rotated slightly, as needed, with regard to the dashed lines) are set as the X and Y axes.
d. Determine the coordinates of the centers of the three regions A, B and C. These are for A (1.203 μm, 2.980 μm), B (4.123 μm, 3.053 μm) and C (5.685 μm, 3.038 μm). The size of each region is set to be 1 μm × 1μm.
e. The same regions are found in each LTEM image as a function of temperature and field.

Supplementary Note 2: Magnitude of external magnetic field applied for LTEM experiments at different temperatures

To perform the magnetic field dependent LTEM experiments under zero-field-cooled (ZFC) and field-cooled (FC) conditions at various temperatures, we recorded the LTEM images at the following magnetic fields (obtained by applying current in the objective lens). Different magnetic fields were used at different temperatures because the nature of the magnetic phase whether helical, anti-skyrmion (aSk) or ferromagnetic (FM), that appears in different parts of the sample, depends on the thickness of the lamella. LTEM images were collected under ZFC (FC) condition, while reducing the magnetic fields after reaching the maximum magnetic fields

of 0.448 (0.429) T, 0.416 (0.384) T, 0.371 (0.362) T, 0.310 (0.320) T, 0.272 (0.278) T and 0.150 (0.157) T at temperatures of 100 K, 150 K, 200 K, 250 K, 300 K and 350 K, respectively.

Supplementary Note 3: Analysis of the anti-skyrmion area of occupation and density

The selected regions from LTEM images were analyzed as follows: after loading the image into a data graphical data analysis program, the center position of the first aSk and the boundary of the same aSk were found manually by clicking on these positions with a mouse. The size of this aSk was thus determined. This same two-click procedure is repeated for all aSks. As shown in Supplementary Figure 2, the aSks are then displayed as red circles. The aSk area ratio is then calculated from the integrated area of the red circles divided by the area of the region. The density of aSk is calculated by dividing the number of aSks by the region size. The size, distance and angles between the aSks are also calculated. The angles are shown in blue in Supplementary Figure 2 and the lines between adjacent aSk in green.

Supplementary Note 4: Example of OOMMF simulation results

In Supplementary Figure 3, the magnetization profile of a 7 layer calculation for B = 2000 Oe is shown. The color corresponds to the z-component of the magnetization, while the black arrows represent the component of the in-plane magnetization. Color scale used here is the same as in the Fig. 5 of the main text. Supplementary Figure 3a-c shows layer 1, 4 and 7 of a simulation of a B20 structure. A clear in-plane angle modulation along the thickness with a specific chirality is clearly seen. Supplementary Figure 3d shows the thickness averaged result: a skyrmion (Sk) lattice is found, but with a reduced in-plane component amplitude. Supplementary Figure 3e-g show layer 1, 4 and 7 of the D_{2d} structure, where no modulation is

found along the thickness direction. Supplementary Figure 3h shows the thickness averaged result of the D_{2d} situation, which are the same as any of the individual layers.

Supplementary Figure 4 shows the magnetization profile of a 13 layer calculation with $B = 2000$ Oe. Supplementary Figure 4a-e shows layer 1, 4, 7, 10 and 13 of the B20 structure, where a clear in-plane angle modulation can be seen along the thickness, and the Sk tube along the z axis is broken. Supplementary Figure 4f shows the thickness averaged result for the B20 situation: due to the breaking of the Sk tube, less contrast is seen and no Sk lattice is found. Supplementary Figure 4g-k show layers 1, 4, 7, 10 and 13 corresponding to a D_{2d} structure, where no modulation of the magnetization is found along the thickness direction. Supplementary Figure 4l shows the thickness averaged result for the D_{2d} situation, where aSk lattice is clearly seen.

In Supplementary Figure 5, we show the magnetization profile of a 13 layer calculation with $B = 300$ Oe. Supplementary Figure 5a-e show layers 1, 4, 7, 10 and 13 of the B20 structure, where a clear in-plane angle modulation is shown along the thickness as a shift of the helix structure. Supplementary Figure 5f shows the thickness averaged result for the B20 situation, in which due to the shift, less contrast is shown and no helix state could be seen. Supplementary Figure 5g-k show Layers 1, 4, 7, 10 and 13 of the D_{2d} structure, where no modulation is shown along the thickness direction. Supplementary Figure 5l shows the thickness averaged result for the D_{2d} situation, where a clear helix structure is found. These results are consistent with the crystal symmetry of a B20 material that has a non-zero DMI component along the z direction whereas a D_{2d} material has a zero component along the z axis.

Supplementary Note 5: Simulation results versus cell size

Simulations with the same parameters but with several cell sizes are performed in order to demonstrate the robustness of the results to the cell size, as illustrated by an exemplary calculation in Supplementary Figure 7. The lattice of anti-Skyrmions that is found under these conditions is not significantly affected by reducing the cell size from 40 nm, which is used otherwise for all the simulations presented in the main and Supplementary Figures 3-6, and 20 nm and 10 nm. The main goal of the simulations that are presented is to demonstrate the distinct thickness dependent phase diagrams that result from the different symmetries of the B20 and D_{2d} crystal structures. The precise details of the simulated magnetic textures will be affected to some extent (as illustrated in Supplementary Figure 7) by the cell size used in the simulations. The cell size used was chosen to make the computations tractable but yet allow the main scientific conclusions to be demonstrated.
